# Real Time 3D Facial Movement Tracking Using a Monocular Camera

**DOI:** 10.3390/s16081157

**Published:** 2016-07-25

**Authors:** Yanchao Dong, Yanming Wang, Jiguang Yue, Zhencheng Hu

**Affiliations:** 1 School of Electronics and Information Engineering, Tongji University, Caoan Road 4800, Shanghai 201804, China; 11wanggyanming@tongji.edu.cn (Y.W.); yuejiguang@tongji.edu.cn (J.Y.); 2 Department of Electrical and Electronics, Kumamoto University, 2-39-1 Kurokami, Kumamoto shi 8608555, Japan; hu@cs.kumamoto-u.ac.jp

**Keywords:** facial animation, facial feature points, 3D facial movement, eyelid, HCI

## Abstract

The paper proposes a robust framework for 3D facial movement tracking in real time using a monocular camera. It is designed to estimate the 3D face pose and local facial animation such as eyelid movement and mouth movement. The framework firstly utilizes the Discriminative Shape Regression method to locate the facial feature points on the 2D image and fuses the 2D data with a 3D face model using Extended Kalman Filter to yield 3D facial movement information. An alternating optimizing strategy is adopted to fit to different persons automatically. Experiments show that the proposed framework could track the 3D facial movement across various poses and illumination conditions. Given the real face scale the framework could track the eyelid with an error of 1 mm and mouth with an error of 2 mm. The tracking result is reliable for expression analysis or mental state inference.

## 1. Introduction

The face image is widely used to discriminate and identify people, in lip reading and to understand one’s emotion and intentions based on the facial expressions [1,2,3]. This paper aims to develop a 3D facial movement tracking framework for real time human computer interface applications such as expression recognition, intention prediction, mental state estimation, etc. In such contexts, the 3D facial movement includes: (a) rigid global head movement and (b) non-rigid facial muscle movement. The rigid global head movement or the head pose consists of a continuous angular measurement of three degree-of-freedom (3-DOF) and a continuous translation measurement of 3-DOF. The non-rigid face movement includes eyelid movement and mouth movement, which play an important role in interpreting the face expression. 

State-of-the-art techniques demonstrate impressive real-time facial animation tracking results by using the depth data from an RGBD camera [4,5]. Video cameras, however, are more widely available on PCs and mobile devices than RGBD cameras, and video-based facial tracking remains a challenging problem. 

The video-based face tracker can be categorized as either motion-based or model-based [6]. The motion-based tracker estimates the displacements of pixels (or blocks of pixels) from one frame to another. The motion field is used to estimate the face motion, but it accumulates motion errors and eventually will lose face tracking. The model-based tracker, on the other hand, uses the prior knowledge of the face structure and motion and tries to update the object’s model parameters to fit new frames. 

Video-based face trackers can also be categorized as either appearance-based or feature-based [6]. Appearance-based trackers match a parameterized model of the entire facial appearance with the test image, trying to find optimal parameters that minimize the distance between the image generated from the synthesized face model and the test image. To search for the optimal parameters in a high-dimensional space brings high computational complexity. Feature-based trackers firstly extract a few facial feature points and then optimize the face pose (and possibly shape) using these feature points. The facial feature points are the fiducial landmarks surrounding facial components: brows, eyes, nose, and mouth. They encode critical information about facial expression and face movement. 

From the perspective of error suppression and computation complexity, the Model-Based & Feature-Based framework is a better choice. For a Feature-Based Tracker the facial landmark localization is a crucial stage.

Active Shape Models (ASM) [7], Active Appearance Models (AAM) [8] and Constrained Local Models (CLM) [9] are classical model based methods for facial landmark localization. In ASM, firstly Procrustes Analysis [10] is applied to the training data to remove similarity transformations, then PCA is utilized to obtain a shape model defined by mean shape and eigenvectors. AAM is an extension of ASM, which contains both the shape statistical model and appearance statistical model. The fitting of AAM can be viewed as an optimization process and Gauss-Newton iterative algorithm is often used to solve this problem. Some modified algorithms have been proposed in the past years to improve the performance of ASM and AAM [11,12,13]. However, most fitting algorithms are sensitive to pose and illumination changes. The initial location in the test image has a significant impact on the result of ASM or AAM, which makes the algorithm less robust. CLM utilizes the same shape model as ASM and AAM, except that the appearance model in CLM is constructed by local facial landmark patches instead of the entire face region. In addition, the appearance model in CLM is used to generate feature templates instead of approximating the image pixels directly. Wang et al. [14] proposed an enforcing convexity strategy at each local patch response surface to optimize a global warp update in an efficient way. This local patch response was obtained by linear SVM and the parametric vector was updated using convex quadratic curve fitting method. Saragih et al. [15] pointed that the effect of ambiguous landmark detections in CLM could be reduced by posing a constraint on joint motion. The non-parametric mean-shift approach was applied over all landmarks simultaneously to impose a global prior over the joint motion. 

Some unified frameworks were proposed to detect the face and landmark simultaneously to handling large facial pose variations. Zhu and Ramanan [16] proposed a unified tree structured part model for face detection, pose and landmark estimation. A mixture-of-trees model was encoded based on the topological changes of viewpoint, and each facial landmark was described by deformable part model. The dynamic programming algorithm was utilized to find global optimal solutions. Yu et al. [17] proposed an optimized part mixtures by using the most significant anchor points while omitting the intermediate landmarks, which reduces the risk of error propagation from misaligned landmarks. However, both the method in [16,17] cannot run in real-time.

In the last few years Discriminative Shape Regression (DSR) has been widely used to locate facial landmarks across different persons, illuminations, and viewpoints in real-time [18,19]. These techniques predict a 2D face shape in a cascade manner: They begin with an initial guess about shape and then progressively refine that guess by regressing a shape update step-by-step from the feature space. Feature spaces can be either global features such as pixel-difference features and Haar-like features in the face region [19,20,21], or local features such as SIFT and local binary features with respect to the landmarks [22,23,24]. The regression function can be either a Ferns regression [19,20], regression trees [21], regression forest [25] or linear regression [22,23,24]. Compared with the model-based methods such as AAM or CLM, the regression based method utilizes the pre-trained regressor to solve the complex, high dimensional, non-linear optimization problem (such as SDM [22]), this regression process is simple and achieves real-time performance. In addition, regression based method directly uses the location of landmarks to describe the face shape.

While most previous face alignment work has emphasized on 2D face tracking and registration, recovering 3D facial movement information from 2D video has not been studied intensively. Only recently [26,27] proposed to recover the 3D face pose and facial animation through regression methodology. Because this kind of approach requires extra scanned 3D face datasets and uses both 2D and 3D annotations, a correction step is needed to resolve inconsistency in the landmark positions across different poses and self-occlusions. 

Inspired by the structure from motion methodology, this paper proposes an analytical approach to estimate 3D facial movement using 2D landmarks in real time. The framework adopts Discriminative Shape Regression to locate the 2D facial feature points on the image and uses an Extended Kalman Filter (EKF) tracking kernel to estimate the 3D facial movement. 

The paper’s main contribution is the development of a framework that combines a 2D facial feature point extraction and a 3D EKF tracking kernel. The framework expands 2D facial landmarks into 3D facial movement information. Given actual face scale the framework could output real world metric. Moreover the framework could register the face shape automatically for different persons.

The arrangement of the paper is as follow: Section 2 presents the algorithm of facial feature point extraction on images; Section 3 gives the 3D face model and its projection model; Section 4 develops the Extended Kalman Filter tracking kernel; Section 5 presents the experiment evaluation of the proposed framework; Section 6 makes a conclusion and gives the future work’s direction.

## 2. Facial Feature Point Extraction

This section presents the discriminative shape regression for facial feature point extraction. We first introduce the training and testing processes of DSR. Then after a quantitative comparison of DSRs with different feature mapping functions and regression functions, we propose to implement the DSR with SIFT feature function and linear regression function since it balances accuracy and real-time processing.

### 2.1. Training

Given N training images ${\left\{I_{i}\right\}}_{i=1}^{N}$ and the corresponding annotated shapes ${\left\{S_{i}^{*}\right\}}_{i=1}^{N}$ with $S_{i}^{*}\in {\mathbb{R}}^{1\times N_{p}}$, where $N_{p}$ is the number of landmarks. The training procedure of DSR can be summarized as following steps, for details please refer to [22,23,24]:

*Step 1*. *Training Data Augmentation*. Each image in the training data is initialized by randomly sampling multiple shapes of other annotated images, the training samples can be expressed by triplets of face image, initial shape estimation and target shape. The triplet can be represented as $\;\left(I_{\pi_{i}},S_{\left(\pi_{i},l\right)}^{\left(0\right)},S_{\pi_{i}}^{*}\right)$, where $\pi_{i}\in \left\{1,\ldots ,N\right\}$ and $S_{\left(\pi_{i},l\right)}^{\left(0\right)}\in \left\{S_{1}^{*},\ldots ,S_{N}^{*}\right\}\backslash S_{\pi_{i}}^{*}$ (l=1,…,L, where L is the number of augmentation). By randomly selecting other annotated shapes as the initial training shapes, one training image can produce different L triplets, this can be viewed as an augmented process, and the total number of these augmented samples is $N_{\text{aug}}=N\times L$.

*Step 2*. *Feature Mapping*. The shape-indexed feature is generated by the feature mapping function $\Phi_{\left(\pi_{i},l\right)}^{\left(t-l\right)}=f\left(I_{\pi_{i}},S_{\left(\pi_{i},l\right)}^{\left(t-l\right)}\right)$, where $\Phi_{\left(\pi_{i},l\right)}^{\left(t-l\right)}\in {\mathbb{R}}^{1\times f}$, f is the feature dimensionality. SIFT features is adopted as the feature mapping function in DSR. It is a kind of local feature that extract only the local region feature coordinated with the facial landmarks. 

*Step 3*. *Regressor Learning*. A regressor in stage t is learned by minimizing the error between the estimated shape $S_{\left(\pi_{i},l\right)}^{\left(t-l\right)}$ and ground truth shape $S_{\pi_{i}}^{*}$ in image $I_{\pi_{i}}$ as:

(1) $$r_{t}=\text{arg min }\overset{N}{\underset{\pi_{i}=1}{\sum }}\overset{L}{\underset{l=1}{\sum }}{\left||{\text{ S}}_{\pi_{i}}^{*}-\left(S_{\left(\pi_{i},l\right)}^{\left(t-l\right)}+r\left(\Phi_{\left(\pi_{i},l\right)}^{\left(t-l\right)}\right)\right)|\right|}^{2}$$

For a linear regressor, r(·) has the form of:

(2) $$r(\Phi_{\left(\pi_{i},l\right)}^{\left(t-l\right)})=\Phi_{\left(\pi_{i},l\right)}^{\left(t-l\right)}\cdot W^{\left(t\right)}+b^{\left(t\right)}=\left[\Phi_{\left(\pi_{i},l\right)}^{\left(t-l\right)},1\right]\cdot \left[W^{\left(t\right)};b^{\left(t\right)}\right]={\tilde{\Phi }}_{\left(\pi_{i},l\right)}^{\left(t-l\right)}\cdot {\tilde{W}}^{\left(t\right)}$$

where ${\tilde{\Phi }}_{\left(\pi_{i},l\right)}^{\left(t-l\right)}=\left[\Phi_{\left(\pi_{i},l\right)}^{\left(t-l\right)},1\right]\in {\mathbb{R}}^{1\times \left(f+1\right)}$ and ${\tilde{W}}^{\left(t\right)}=\left[W^{\left(t\right)};b^{\left(t\right)}\right]\in {\mathbb{R}}^{\left(f+1\right)\times 2N_{p}}$. Then, Equation (2) becomes a least squares problem and has a closed solution as:

(3) $${\tilde{W}}^{\left(t\right)}={\left[{\left({\tilde{\Phi }}^{\left(t-l\right)}\right)}^{T}\cdot {\tilde{\Phi }}^{\left(t-l\right)}+\lambda E\right]}^{-1}\cdot {\left({\tilde{\Phi }}^{\left(t-l\right)}\right)}^{T}\cdot {\Delta S}^{\left(t-l\right)}$$

(4) $$r_{t}\left({\tilde{\Phi }}^{\left(t-l\right)}\right)={\tilde{\Phi }}^{\left(t-l\right)}\cdot {\tilde{W}}^{\left(t\right)}$$

where ${\tilde{\Phi }}^{\left(t-l\right)}={\left\{\left[\Phi_{\left(\pi_{i},l\right)}^{\left(t-l\right)},1\right]\right\}}_{\pi_{i}=1,\ldots ,N;l=1,\ldots ,L}\in {\mathbb{R}}^{N_{\text{aug}}\times \left(f+1\right)}$ is the feature matrix, $E\in {\mathbb{R}}^{\left(f+1\right)\times \left(f+1\right)}\;$ is the identity matrix, λE is used to avoid the inversion of singular matrix and ${\Delta S}^{\left(t-l\right)}={\left\{S_{\pi_{i}}^{*}-S_{\left(\pi_{i},l\right)}^{\left(t-l\right)}\right\}}_{\pi_{i}=1,\ldots ,N;l=1,\ldots ,L}\in {\mathbb{R}}^{N_{\text{aug}}\times 2N_{p}}$ is the error matrix between the ground truth shapes and the current estimated shapes.

*Step 4. Shape Update.*

(5) $$S^{\left(t\right)}=S^{\left(t-1\right)}+r_{t}\left({\tilde{\Phi }}^{\left(t-l\right)}\right)$$

where $S^{\left(t-1\right)}={\left\{S_{\left(\pi_{i},l\right)}^{\left(t-l\right)}\right\}}_{\pi_{i}=1,\ldots ,N;l=1,\ldots ,L}\in {\mathbb{R}}^{N_{\text{aug}}\times 2N_{p}}$.

Step 2–Step 4 iterate in a gradient boosting framework until $S^{\left(t\right)}$ converged to the target shapes. 

### 2.2. Runtime Regreesion

Given a new image I with an initial shape $S^{\left(0\right)}$, the landmark is regressed by the learned cascaded regressor $r_{t}$ stage by stage as:

(6) $$S^{\left(t\right)}=S^{\left(t-1\right)}+r_{t}\left(f\left(I,S^{\left(t-1\right)}\right)\right)$$

where I is the input image, $S^{\left(t\right)}={\left[x_{1}^{\left(t\right)},x_{2}^{\left(t\right)},\ldots ,x_{N_{p}}^{\left(t\right)},y_{1}^{\left(t\right)},y_{2}^{\left(t\right)},\ldots ,y_{N_{p}}^{\left(t\right)}\right]}^{T}$ is the shape with $N_{p}$ facial landmarks in I at stage t, f(·)is the shape-indexed feature mapping function depends on both image I and previous estimated shape $S^{\left(t-1\right)}$, $r_{t}\;$ is the regression function in stage t and t=1,…,T is the number of cascade level.

The success of discriminative regression method is mainly due to the following properties: (1) the shape-indexed feature in each stage makes a re-sampling at the previous estimation of the landmark location. This feature extract method compensates the effect of large appearance variations and increases the robustness and accuracy; (2) gradient boosting framework is incorporated in the training procedure of regression functions. In each stage the regression function is learned based on the previously estimated shape error and the shape-indexed feature. Thus, the output error in each stage monotonically decreases and converges in 4 or 5 stages; (3) the output of the regressor is a linear combination of training shapes which inherently guarantees the output is a reasonable face shape without any extra constraints.

### 2.3. Feature Mapping Functions and Regression Functions

The feature mapping and regressor learning are the two main steps in DSR. The selection of feature mapping function and regression function may affect the DSR performance. In this section, we compare different DSR implementations to find a better one. Candidate feature mapping functions include pixel-difference features and SIFT features and candidate regression functions include ferns regression, regression trees and linear regression. The LFPW dataset [28] and COFW dataset [20] are utilized to make a quantitative comparison.

Pixel-difference Features (PFs) calculate the intensity difference of F (here F=400 in our implementation) pairs of pixels in the face region. These pixel intensities are obtained by interpolated shape-indexed features, the correlation-based feature selection is utilized to select the final features (128 dimensions in our paper) for regression. SIFT Features (SFs) are obtained by resizing the width of face in the training image into 255 pixel width and then a pixel region around each facial landmark is used to extract SIFT features. The feature dimensions of SFs for a single landmark is 128. The final features are composed by the cascade of these local SIFT features and with the dimension of $128\times N_{p}$. 

Ferns Regressor (FR) is implemented with depth of 5 and two-level cascaded regression with T = 100 and K = 50. T is the number of cascade level and K is the number of regressors that used in each cascade level. Trees Regressor (TR) is with depth of 5 and the minimum samples for each leaf node is set to 5, two-level cascaded regression is adopted with T = 10 and K = 10 similar as ferns regressor. Linear Regressor (LR) is utilized as described in Section 2.1 with T = 5 in LFPW dataset and T = 10 in COFW dataset for more complex environment.

**Figure 1:**
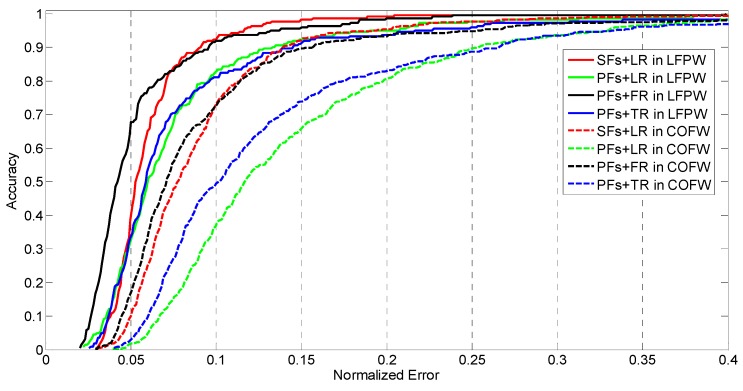
The comparison of CED curves on LFPW and COFW datasets for different combinations of feature mapping function and regression function.

All the datasets for the experiments are initialized with L = 20 and the initial shape is selected as the mean shape for testing. Actually, SFs has high-dimension feature space ($128\times N_{p}$) and not suitable for ferns and tree regression, so we didn’t implement DSR with these combinations. Figure 1 shows the CED curve of these comparisons.

Figure 1 shows that the combination of PFs and FR gives the best performance. This method uses simple features but large number of weak regressors (5000 in our application) to obtain good results. However, its computation cost is high. TR is stronger than ferns and linear regression. Actually, ferns can be viewed as a simplified version of tree regression with randomly split features chosen. The number and depth of TR have significant effect on the regression result, which cause the regressor tend to be under-fitting or over-fitting. SFs contain more information (such as gradient and directional information) but its computation cost is higher than PFs. However, this strong features need fewer iterations for shape regression (T=5 in LFPW and T=10 in COFW), which decreases the computation time of regressor. Considering the balance of accuracy and computation efficiency, we propose to implement DSR with SIFT features and linear regression.

## 3. Projection Model

Give the 3D face model a projection model is required to transform 3D face onto 2D images. The relevant transformations and the associated variables are:

(1) Local geometry transformation of face shape and animation parameters (associated variables are $\vec{s}$ and $\vec{a}$),
(2) Scaling, rotation and translation of the face in the world coordinate system (associated variables are $\vec{c},\vec{r}$ and $\vec{t}$),
(3) Rotation and translation of the world coordinate system in the camera coordinate system,
(4) Projection on the image with the generalized camera projection matrix.

### 3.1. Face Model

**Figure 2:**
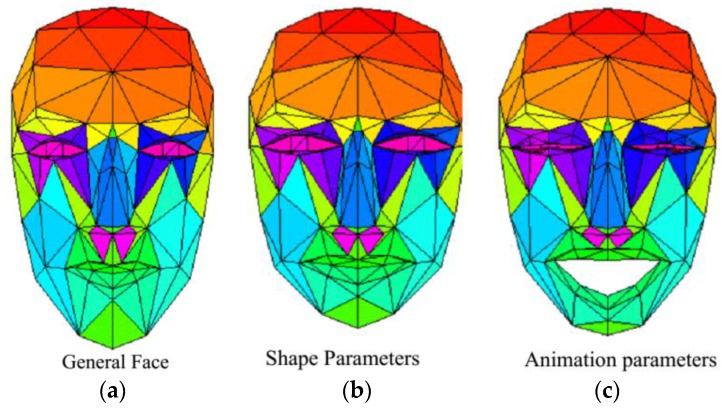
Candide-3 face model. (**a**) Is the general face; (**b**) is a face modified by shape parameters; (**c**) is a face modified by shape and animation parameters.

In the past decades, several face models have been created by different research groups. Some use thousands of polygons to describe a face, and some model the complex underlying physics. A 3D face model is needed to deal with the task of 3D face pose and facial animation tracking. The paper adopts the Candide-3 [29] face model because its simplicity makes it a good tool for image analysis. Candide-3 is controlled by global and local Action Units (AUs). The global AUs correspond to scaling, rotations and translation along three axes. The local AUs control the mimics of the face so that different expressions can be produced. It contains 113 vertices which are connected by lines forming 184 triangular surfaces, as shown in Figure 2.

### 3.2. Transformations

#### 3.2.1. Local Geometry Transformation

The local geometry (shape, structure) is determined by the 3D coordinates of the vertices. Candide-3 implements a set of shape units and animation units which control the face’s shape and animation respectively. Let $V^{1}=\left[\vec{s},\;\vec{a}\right]$ be the shape and animation parameters and ℒ the corresponding transformation. Then in model-centered coordinate system ℒ is defined as:

(7) $$b_{m_{i}}={\bar{g}}_{i}+S_{i}\cdot \vec{s}+A_{i}\cdot \vec{a}$$

where i indicates the ith vertex in the face model, $\bar{g}$ is the general face model, the columns of S and A are the Shape Units and Animation Units, respectively, and the column vector $\vec{s}$ and $\vec{a}$ contain the shape and animation variables. By adjusting the shape and animation parameters ($\vec{s}$ and $\vec{a}$) the face model $b_{m}$ could fit to any person and any facial expression.

#### 3.2.2. Global Transformation

Global transformation parameters define the face scaling, rotation and translation in the world coordinate system. Let $V^{2}=\left[c_{x},{\text{ c}}_{y},{\text{ c}}_{z},{\text{ r}}_{x},{\text{ r}}_{y},{\text{ r}}_{z},{\text{ t}}_{x},{\text{ t}}_{y},{\text{ t}}_{z}\right]$ be global parameters and G the corresponding transformation, where $c_{x},{\text{ c}}_{y},{\text{ c}}_{z}$ are the scaling factors in the *x*-, *y*- and *z*-axis; $r_{x},{\text{ r}}_{y},{\text{ r}}_{z}$ are rotation angles around the *x*-, *y*- and *z*-axis;${\text{ t}}_{x},{\text{ t}}_{y},{\text{ t}}_{z}$ are translations along the *x*-, *y*- and *z*-directions. Then G is defined as:

(8) $$b_{w_{i}}=R\cdot C\cdot b_{m_{i}}+\vec{t}=R\cdot C\cdot \left({\bar{g}}_{i}+S_{i}\cdot \vec{s}+A_{i}\cdot \vec{a}\right)+\vec{t}$$

where $b_{w_{i}}$ is the coordinate of the ith face vertex in the world coordinate system, R is the rotation matrix $R=R\left(r_{x},{\text{ r}}_{y},{\text{ r}}_{z}\right)$, C is the scaling matrix $C=\text{diag}\left(c_{x},{\text{ c}}_{y},{\text{ c}}_{z}\right)$, $\vec{t}$ is the translation vector $\vec{t}=\left[t_{x},{\text{ t}}_{y},{\text{ t}}_{z}\right]$.

#### 3.2.3. Camera Transformation

Denote $b_{w_{i}}={\left[x_{w_{i}},{\text{ y}}_{w_{i}},{\text{ z}}_{w_{i}}\right]}^{T}$ as a point in the world coordinate system, $R_{wc}$ and $t_{wc}$ are the rotation matrix and translation vector of the world coordinate system with respect to the camera coordinate system. Note T as the transformation of point X from world coordinate system to camera coordinate system then T is defined as:

(9) $$b_{c_{i}}={\left[x_{c_{i}},{\text{ y}}_{c_{i}},z_{c_{i}}\right]}^{T}=\;R_{wc}\cdot b_{w_{i}}+t_{wc}$$

#### 3.2.4. Camera Model

A standard pin-hole model was used for the generalized camera projection. For a calibrated camera there is no unknown. Let P be the projection of a point in camera coordinate system to the image coordinate system and $p_{i}$ as the projected image point, then in the pin-hole model P is defined as:

(10) $$p_{i}={\left(u_{i},v_{i}\right)}^{T}=\;\emptyset \left(A_{cam},b_{c_{i}}\right)=\;{\left(f_{x}\frac{x_{c_{i}}}{z_{c_{i}}}+u_{0},f_{y}\frac{y_{c_{i}}}{z_{c_{i}}}+v_{0}\right)}^{T}$$

where $A_{cam}$ is the matrix representing the camera’s intrinsic parameters: the principle point $\left(u_{0},{\text{ v}}_{0}\right)$, the focus in u- and v-axis $\left(f_{x},{\text{ f}}_{y}\right)$.

#### 3.2.5. Final Equations

Let ℱ be the composition of different projections and Let V be the wanted unknown parameters, the projection model is:

(11) F=P∘T∘G∘L

(12) $$V=\left[V^{1},V^{2}\right]=\left[\vec{s},\;\vec{a},\vec{c},\vec{r},\vec{t}\right]=\left[\vec{s},\;\vec{a},c_{x},{\text{ c}}_{y},{\text{ c}}_{z},{\text{ r}}_{x},{\text{ r}}_{y},{\text{ r}}_{z},{\text{ t}}_{x},{\text{ t}}_{y},{\text{ t}}_{z}\right]$$

The pose of the face is represented by $\vec{r}$ and $\vec{t}$, the face shape is represented by $\vec{c}$ and $\vec{s}$, and the face animation by $\vec{a}$. So the 3D facial tracking problem now becomes estimating the value of vector V. Respectively, to track face pose and animation is to estimate $\vec{r}$**,** $\vec{t}$ and $\vec{a}$; while parameters $\vec{c}$ and $\vec{s}$ define the scale and the shape of the face for different persons.

## 4. Tracking Kernel

Given N facial feature points $p_{i}$ obtained from the facial feature point extraction module and their corresponding vertex in the face model $b_{mi}$, the 3D facial movement tracking problem can be solved by minimizing the following function:

(13) $$F\left(V\right)=\frac{1}{2}\sum_{i=1}^{N}{\left[ℱ\left(V,b_{m_{i}}\right)-p_{i}\right]}^{2}$$

This cost function minimizes the Euclidean distance between the projected points of the model vertexes and the extracted points in the image. 

Normally different persons have different face shapes and the face would take random global or local motions. So every parameter in vector V is a random variable. But the evolution of vector V from time $t_{k}$ to time $t_{k+1}$ can be approximated by some classical dynamic model plus additive noise. Here a zero-order evolution model is adopted, where the object’s motion is considered as static evolution. The state evolution equation is:

(14) $$x_{k+1}=A_{e}\cdot x_{k}+M\cdot w_{k}$$

where x is the state vector and is defined as x≡V, $x_{k+1}$ and $x_{k}$ is the value of the state vector at time $t_{k+1}$ and $t_{k}$, $A_{e}$ is an identity matrix, M equals to the sampling time $T_{s}$, and the process noise $w_{k}$ is a zero-mean Gaussian white noise vector. Assume the initial state error covariance matrix is $P_{0}$, the noise distribution satisfy $p\left(w_{k}\right)∼N\left(0,Q_{k}\right)$ and $p\left(v_{k}\right)∼N\left(0,R_{k}\right)$, $Q_{k}$ is the covariance matrix of process noise and $R_{k}$ is the covariance matrix of measurement noise. The paper utilizes the Extended Kalman Filter (EKF) algorithm to recursively solve the facial movement problem as follow:

Firstly, predict the a priori of state vector and its covariance matrix at $t_{k+1}$ using the evolution model as Equation (15a,b):

(15a) $$x_{k+1}^{-}=A_{e}\cdot x_{k}^{+}$$

(15b) $$P_{k+1}^{-}=A_{e}\cdot P_{k}^{+}\cdot A_{e}^{T}+M\cdot Q_{k}\cdot M^{T}$$

where $x_{k}^{+}$ is the posterior state vector value at time $t_{k}$, $x_{k+1}^{-}$ is the prior state vector value at time $t_{k+1}$, $P_{k}^{+}$ is the posterior covariance matrix of state error at time $t_{k}$, $P_{k+1}^{-}$ is the prior covariance at time $t_{k+1}$, $Q_{k}$ is the covariance matrix of process noise at time $t_{k}$.

In Equation (14), a zero-order static evolution system model is adopted to approximate the face movement dynamics. There exist some modeling errors for the approximation inevitably, which would result inaccuracy estimation. To compensate these modeling errors the fading-memory filter is introduced. The fading-memory filter is identical to the standard Kalman filter, except that the prediction of $P_{k+1}^{-}$ has a factor $\alpha^{2}$ in its first term as in Equation (15c). This factor serves to increase the uncertainty in the state prediction, which gives more credence to the measurement. In most applications, α is only slightly greater than 1 (for example, 1.01):

(15c) $$P_{k+1}^{-}=\alpha^{2}\cdot A_{e}\cdot P_{k}^{+}\cdot A_{e}^{T}+M\cdot Q_{k}\cdot M^{T}$$

When new measurement arrives we have to correct the a priori using the new measured facial feature points $\bar{p}$ ($\bar{p}=\left[p_{0},\;p_{1},\cdots \;,p_{N_{p}}\right]$ is the vector of the measured facial feature points, $N_{p}$ is the total number of feature points). Given the a priori we could derive the facial feature points using the projection model $ℱ\left(x_{k+1}^{-},{\bar{b}}_{m}\right)$ as in Equation (11), where ${\bar{b}}_{m}=\left[b_{m_{0}},\;b_{m_{1}},\cdots \;,b_{m_{N_{p}}}\right]$ is the corresponding vertex of $\bar{p}$ in the basic face model. The difference between the measured points $\bar{p}$ and the projected points $ℱ\left(x_{k+1}^{-},{\bar{b}}_{m}\right)$ is used as the residual to correct the predicted a priori: $x_{k+1}^{-}$ and $P_{k+1}^{-}$. The correction equation is (16a) and (16b):

(16a) $$x_{k+1}^{+}=x_{k+1}^{-}+K\cdot \left(\bar{p}-ℱ\left(x_{k+1}^{-},{\bar{b}}_{m}\right)\right)$$

(16b) $$P_{k+1}^{+}=\left(I-K\cdot J_{hx}\right)\cdot P_{k+1}^{-}$$

where $x_{k+1}^{+}$ is the posterior state vector at time $t_{k+1}$, $P_{k+1}^{+}$ is the posterior covariance matrix of state error at time $t_{k+1}$, K is the Kalman gain factor and $J_{hx}$ is the Jacobean matrix of measurement to state vector. 

The state error covariance matrix P should be a symmetric positive definite matrix. Because of numerical computing problems, the posterior update of covariance matrix $P_{k+1}^{+}$ in Equation (16b) does not guarantee $P_{k+1}^{+}$ is positive definite even when $P_{k+1}^{-}$ and $P_{k}^{+}$ are positive definite. So Equation (16b) is replaced with Equation (16c), which guarantees $P_{k+1}^{+}$ is positive definite:

(16c) $$P_{k+1}^{+}=\left(I-K\cdot J_{hx}\right)\cdot P_{k+1}^{-}\cdot {\left(I-K\cdot J_{hx}\right)}^{T}+K\cdot R_{k}\cdot K$$

The Kalman gain factor K is obtained using Equation (17):

(17) $$K=P_{k+1}^{-}\cdot J_{hx}^{T}\cdot {\left(J_{hx}\cdot P_{k+1}^{-}\cdot J_{hx}^{T}+R_{k}\right)}^{-1}$$

Observing Equations (16b,c) and (17) it is easy to find that $P_{k+1}^{-}$ is already available from the a priori, $R_{k}$ can be determined from the facial feature point extraction module as covariance of measurement noise, $J_{hx}$ is the Jacobean matrix of measurement to state vector. Equation (11) is the measurement model for Kalman filter, which project the state vector to the facial feature points. Since the projecting process is nonlinear we have to linearize the measurement model at the current state point and compute the $J_{hx}$ at every time step, which is called Extended Kalman Filter. The Jacobean matrix is defined as:

(18) $$J_{hx}\equiv \frac{\partial \bar{p}}{\partial x_{k+1}^{-}}=\frac{\partial \left[p_{0},\;p_{1},\cdots \;,p_{N_{p}}\right]}{\partial x_{k+1}^{-}}={\left[\frac{\partial p_{0}}{\partial x_{k+1}^{-}},\frac{\partial p_{1}}{\partial x_{k+1}^{-}},\cdots \;,\frac{\partial p_{N_{p}}}{\partial x_{k+1}^{-}}\right]}^{T}$$

Using the chain rule of partial differential, $\frac{\partial p_{i}}{\partial x_{k+1}^{-}}$ can be written as:

(19) $$\frac{\partial {\bar{p}}_{i}}{\partial x_{k+1}^{-}}=\frac{\partial {\bar{p}}_{i}}{\partial {\left[\vec{s},\;\vec{a},\vec{c},\vec{r},\vec{t}\right]}_{k+1}^{-}}=\left[\frac{\partial p_{i}}{\partial b_{c_{i}}}\cdot \frac{\partial b_{c_{i}}}{\partial b_{w_{i}}}\cdot \left[\frac{\partial b_{w_{i}}}{\partial b_{m_{i}}}\cdot \left[\frac{\partial b_{m_{i}}}{\partial \vec{s}},\frac{\partial b_{m_{i}}}{\partial \vec{a}}\right],\frac{\partial b_{w_{i}}}{\partial \vec{c}},\frac{\partial b_{w_{i}}}{\partial \vec{r}},\frac{\partial b_{w_{i}}}{\partial \vec{t}}\right]\right]$$

$\frac{\partial p_{i}}{\partial b_{c_{i}}}$ can be derived from Equation (10) as:

(20a) $$\frac{\partial p_{i}}{\partial b_{c_{i}}}=\left[\begin{array}{c} \frac{\partial u_{i}}{\partial b_{c_{i}}}\\ \frac{\partial v_{i}}{\partial b_{c_{i}}} \end{array}\right]=\left[\begin{array}{ccc} \frac{f_{x}}{z_{c_{i}}} & 0 & -\frac{f_{x}\cdot x_{c_{i}}}{z_{c_{i}}^{2}}\\ 0 & \frac{f_{y}}{z_{c_{i}}} & -\frac{f_{y}\cdot y_{c_{i}}}{z_{c_{i}}^{2}} \end{array}\right]$$

$\frac{\partial b_{c_{i}}}{\partial b_{w_{i}}}$ can be derived from Equation (9) as:

(20b) $$\frac{\partial b_{c_{i}}}{\partial b_{w_{i}}}=R_{wc}$$

$\frac{\partial b_{w_{i}}}{\partial b_{m_{i}}}$, $\frac{\partial b_{w_{i}}}{\partial \vec{c}}$, $\frac{\partial b_{w_{i}}}{\partial \vec{r}}$ and $\frac{\partial b_{w_{i}}}{\partial \vec{t}}$ can be derived from Equation (8) as:

(20c) $$\frac{\partial b_{w_{i}}}{\partial b_{m_{i}}}\;=R\cdot C$$

(20d) $$\frac{\partial b_{w_{i}}}{\partial \vec{c}}\;=R\cdot \left[\begin{array}{ccc} x_{m_{i}} & & \\ & y_{m_{i}} & \\ & & z_{m_{i}} \end{array}\right]$$

(20e) $$\frac{\partial b_{w_{i}}}{\partial \vec{r}}\;=\frac{\partial R}{\partial \vec{r}}\cdot b_{m_{i}}=\left[\begin{array}{ccc} \frac{\partial R}{\partial r_{x}} & \frac{\partial R}{\partial r_{y}} & \frac{\partial R}{\partial r_{z}} \end{array}\right]\cdot b_{m_{i}}$$

(20f) $$\frac{\partial b_{w_{i}}}{\partial \vec{t}}\;=I_{3\times 3}$$

$\frac{\partial b_{m_{i}}}{\partial \vec{s}}$ and $\frac{\partial b_{m_{i}}}{\partial \vec{a}}$ can be derived from Equation (7) as:

(20g) $$\frac{\partial b_{m_{i}}}{\partial \vec{s}}\;=S_{i}$$

(20h) $$\frac{\partial b_{m_{i}}}{\partial \vec{a}}\;=A_{i}$$

Substituting Equation (20a–h) into Equation (19) yields the partial differential of one facial feature point to the state vector. Then the Jacobean matrix of Equation (18) can be obtained by padding all the partial differential of the measured feature points along the row. Jacobean matrix should be computed every step using the a priori value. 

Given Equations (15)–(18) we could estimate the face 3D movement recursively using newly measured facial feature points.

During implementation the pose ($\vec{r},\vec{t}$), shape ($\vec{s},\;\vec{c}$) and animation ($\vec{a}$) parameters are optimized using an alternating strategy. Firstly the pose is optimized then the shape is optimized and finally the animation is optimized. The alternating strategy could reduce the coupling problem between the parameters and fit to different persons more accurately.

## 5. Experimental Evaluations

The framework is implemented on a Windows 8 PC using C++ and runs in real time. The DSR algorithm for facial feature point (FFP) extraction is trained with two widely used datasets: Labeled Face Parts in the Wild (LFPW) [28] and Caltech Occluded Faces in the Wild (COFW) [20]. Among the localized landmarks a sparse set of them is used for 3D facial movement tracking: *inner points of brows, inner points and outer points of eyes, middle points* *of* *upper and lower eyelids, nose tip, left and right corners of the mouth and middle points of upper and lower lip*. With these Facial Feature Points (FFPs) as input the tracking kernel could estimate the face pose, eyelid and mouth movement.

### 5.1. Rotation Tracking

Firstly, we use the Boston University Face Tracking (BUFT) dataset [30] to evaluate the performance of our pose estimation method. BUFT dataset has 45 video sequences recorded from 5 testers, each video has about 200 frames, each frame has resolution of 320 × 240, and the rotations of yaw, roll and pitch are provided as ground truth. In the literature the magnetic tracker or 3D inertia tracker is chosen as ground truth but it should be pointed out that they are not the real ground truth but the approximations [31]. What’s more, the inertia sensor has error accumulation problem.

The proposed tracking kernel in Section 4 is utilized to estimate the 3D head orientation in each video, the Mean Absolute Error (MAE) between the estimated values and ground truths are given in Table 1. Some comparisons of MAE with other state-of-arts 3D pose tracking algorithms are also given in Table 1.

Table 1 shows that our proposed method has the comparable if not the minimum mean error than other algorithms. The algorithm in [15] is realized with 3D CLM model and considers the joint motion of each landmark, it has the similar MAE with our proposed method, however, this algorithm requires a recalibration procedure using the ground truth when a large drift occurs, which is infeasible in the real environment.

To further evaluate the performance of our framework, we recorded a set of test videos in lab environment using a higher resolution camera. A magnetic compass sensor is placed onto the tester’s head and used as reference for head rotation. The compass sensor outputs the yaw, roll and pitch values by sensing the earth magnetic field. The MAE between the estimated values and compass sensor outputs are calculated for different testers, and the result is given in Table 2.

**Figure 3:**
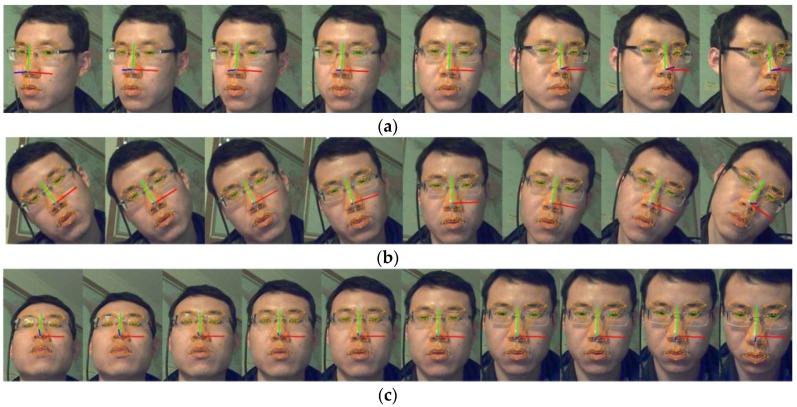
Images of rotation tracking results. (**a**) Is the images of yaw tracking; (**b**) is the images of roll tracking and (**c**) is the images of pitch tracking.

The accuracy of the compass sensor is 1.5°, 0.2° and 0.2° for the yaw, roll and pitch respectively. The working range of the rotation is [−35°, +35°], [−35°, +35°] and [−25°, +25°] for yaw, roll and pitch. The MAE between our result and the compass value is 2.8°, 2.3° and 2.2° for yaw, roll and pitch respectively. If the working range is controlled within the range of [−15°, +15°] then the MAE is 2.4°, 2.0°, 2.1°. Figure 3a–c show sample face images of yaw, roll and pitch. The face coordinate system on the image represents the rotation parameters graphically.

The roll tracking result is the best one among the three rotations. Because roll is in-plane-rotation, there is no FFPs lost and coupling problem during the tracking. Within the range of [−15°, +15°] the yaw tracking obtains good performance with an MAE of 2.4°, while beyond the range the yaw tracking error increases dramatically. The reason is that some FFPs will become self-occluded for large yaw rotation and the FFPs extraction error will increase. The precision of pitch tracking is not as good as roll and yaw, that is because the pitch parameter is coupled with other parameters, say $t_{y}$.

### 5.2. Eyelid and Mouth Movement Tracking

#### 5.2.1. Database Evaluation

There is no public released database particularly designed for evaluating the eyelid and mouth tracking. In [35] the authors recorded videos to test the blinking hit rate but the dataset is not open. In this paper we use the 300 Videos in the Wild (300-VW) database to evaluate our algorithm. The 300-VW dataset has is released by iBUG group of Imperial College London. This dataset aims at testing the ability of fitting unseen subjects, independently of variations in pose, expression, illumination, background, occlusion, and image quality. The videos can be categorized into three scenarios. Scenario 1 contains a number of testing videos will be of people recorded in well-lit conditions displaying arbitrary expressions in various head poses; Scenario 2 contains a number of testing videos will be of people recorded in unconstrained conditions displaying arbitrary expressions in various head poses but without large occlusions; Scenario 3 contains a number of testing videos will be of people recorded in completely unconstrained conditions including the illumination conditions, occlusions, make-up, expression, head pose, etc. Scenario 3 mainly aims to assess the performance of facial landmark detection and tracking in arbitrary conditions, while our algorithms focus on 3D face movement tracking. Hence, we choose 31 videos without large occlusion from scenarios 1 and 2 to test our algorithm. There are about 1800 frames for per video and each frame has been annotated manually using the 68 points mark-up. 

We projected the tracked 3D face model back to the image and calculate the eyelid distance, mouth width and mouth height in pixel. These parameters can also be derived from the manually annotated 68 points, which is treated as the ground truth. Then the mean absolute error and standard deviation can be computed for each video. Table 3 lists some videos’ result. The maximum mean absolute error (MAE) of left eyelid distance is 2.2 pixels happened in video ”123”; The maximum MAE of right eyelid distance is 2.2 pixels happened in video ”143”; The maximum MAE of mouth width is 4.4 pixels happened in video ”019”; And the maximum MAE of mouth height is 3.4 pixels happened in video ”037”. Since the face size in the image varies Table 3 also gives the pupil distance in pixel as an indicator of the face size. When comparing different videos the face size should be taken into count.

**Figure 4:**
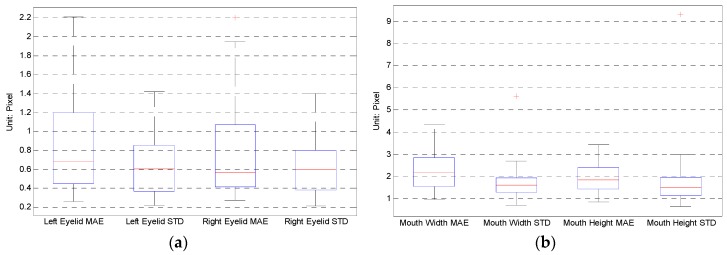
Boxplot of the mean absolute error (MAE) and standard deviation (STD) of the 31 videos. (**a**) Boxplot of eyelid distance MAE and STD; (**b**) boxplot of mouth width and height MAE and STD.

Figure 4 gives the boxplots of eyelid and mouth MAE and STD of the 31 chosen videos. Figure 4a shows that the MAE of the eyelid distance mainly locates within 1.2 pixels, while Figure 4b shows that the MAE of the mouth width and height mainly locate within 2.8 pixels.

**Figure 5:**
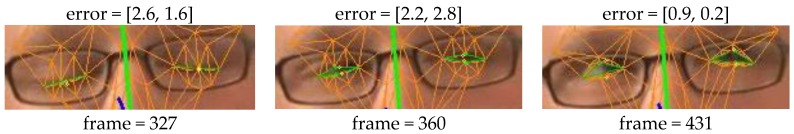
Eyelid distance errors for images from video ”123”. The yellow point is the ground truth, the greed point is the tracked result. The error is defined as absolute difference between the tracked eyelid distance and the ground truth. Take frame 327 for example, the left eyelid error is 2.6 pixels while the right eyelid error is 1.6 pixels. The pupil distance is 101.1 pixels for video ”123”.

**Figure 6:**
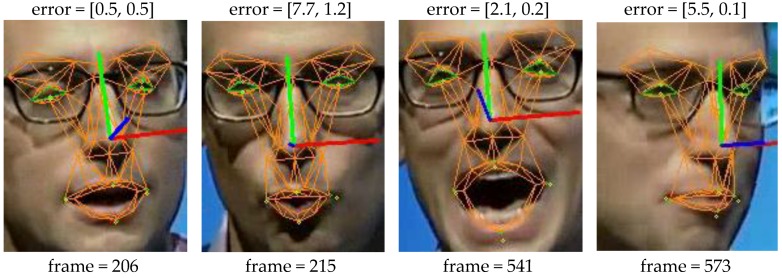
Mouth width and height errors for images from video ”019”. The yellow point is the ground truth, the greed point is the tracked result. The error is defined as absolute difference between the tracked mouth width (height) and the ground truth. Take frame 206 for example, the mouth width error is 0.5 pixels while the mouth height error is 0.5 pixels. The pupil distance is 74.8 pixels for video ”019”.

Figure 5 and Figure 6 give more direct interpretation about the tracking result. Video “123” has the biggest eyelid tracking error while the video “019” has the biggest mouth tracking error as shown in Table 3. Figure 5 shows the eyelid tracking result of video ”123”. The tester wears a pair of glasses and the image contrast is not very well. The eyelid edge is not very sharp which poses challenge to the facial feature point extraction. That is why this video shows biggest eyelid tracking error. Figure 5 shows three cropped images of eye region from the video ”123”, their eyelid tracking errors are shown above the images. Though the error may reach 2.6 pixels it is still small compared to the eye size, and it is reliable to derive the eyelid motion behaviour using the tracked result. Figure 6 shows the mouth tracking result of video ”019”. The tester performs an indoor speech and the poses and expression varies strongly. Besides, there is obvious illumination problem and shadows are casted onto the face. Though mouth width tracking error in frame 215 and 573 is larger, which is mainly caused by shadow and self-occlusion, it is still obvious that the mouth model tracked the image correctly across expression and pose variations.

#### 5.2.2. Real Metric Evaluation

300-VW database annotates the facial feature points on the image as ground truth, their unit is pixel. For some real world applications the real metric is wanted rather than pixel. But for the face pose and animation estimation, it is impossible to get the real metric ground truth for all of the state variables no matter what kind of sensor is utilized. In order to obtain the real metric rather than pixel we register the face by fusing its actual size. And the manually measured distance in real metric is used as an approximation of ground truth.

**Figure 7:**
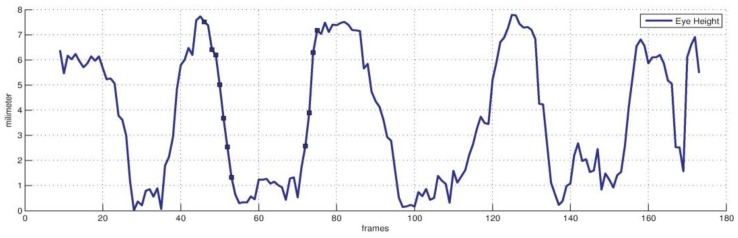
Eyelid distance curve in metric of millimetre.

**Figure 8:**
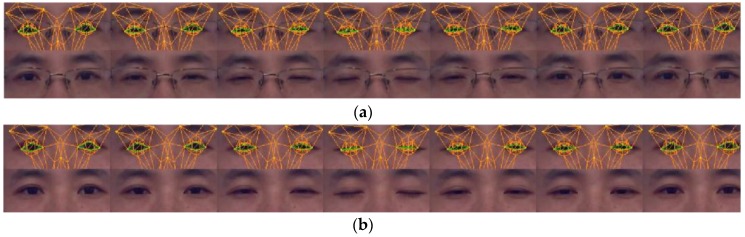
Sample images of eye movement tracking. The tracked eyelid is drawn by green lines. (**a**) Wears a pair of glasses and (**b**) wears no glasses.

Figure 7 and Figure 8 show the result of eyelid tracking. Figure 7 shows the curve of tracked eyelid distance in a recorded video. The eyelid distance is an important parameter for determining eye and mental status. It corresponds to the *eye closure* variable in animation vector $\vec{a}$. This variable has no unit so it should be synthesized with the registered scaling factor $\vec{c}$ and the 3D face model to yield the eyelid distance with millimetre as the metric unit. The curve in Figure 7 corresponds to a sequence of blinks. It shows that the eyelid distance varies between 0 mm and 8 mm and the maximum distance is 7.8 mm. The manually measured eyelid distance of the tested person is 8 mm when the eye is open naturally. The dark points on the curve are the sampled instances during a typical blinking procedure and their corresponding images are listed in Figure 8a. The raw eye region is shown in the lower row and the overlaid image by tracked eye model is shown in the upper row. The green lines are the estimated eyelid. Figure 8 shows that the estimated eyelid model tracks the real eyelid correctly for various openness degrees. Figure 8b shows similar samples as Figure 8a excepting that the tester in Figure 8b wears no glasses. Figure 7 and Figure 8 show that the proposed tracker could estimate the eyelid distance correctly with an accuracy of less than 1 mm.

**Figure 9:**
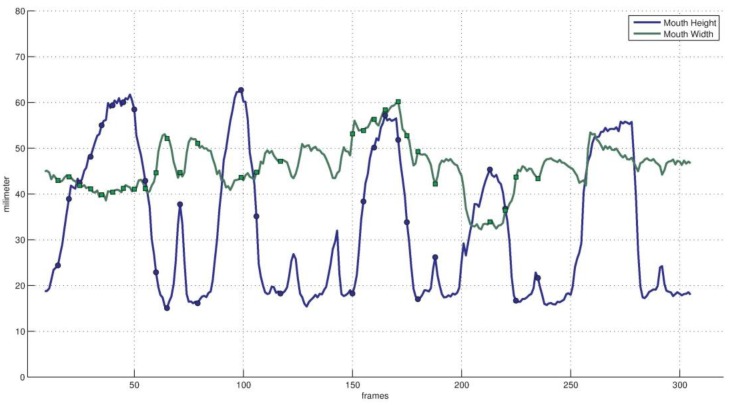
Mouth movement curves in metric of millimetre. The green curve is the mouth width tracking result while the blue curve is the mouth height tracking result.

**Figure 10:**
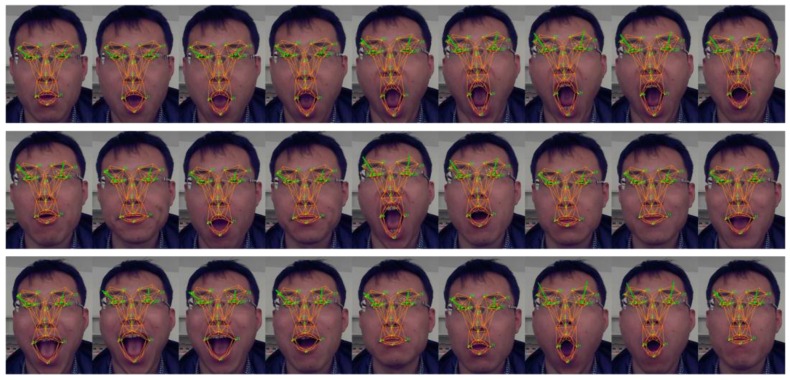
Sample images of mouth movement tracking. Their corresponding mouth metrics are the dot points on the curves in Figure 9.

Figure 9 and Figure 10 show the result of mouth tracking. Figure 9 shows the curves of mouth *width* and *height* in a recorded video. Mouth movement is determined by three variables in animation vector $\vec{a}$: *mouth stretch*, *upper lip raise* and *jaw drop*. *Mouth stretch* tracks the movement of mouth corners, *upper lip raise* tracks the movement of upper lip and *jaw drop* tracks the movement of lower lip and jaw. Hence, the corners of the mouth and the middle points of the upper and lower lips are used to estimate these variables. Mouth movement plays an important role in interpreting facial activity. As the *eye closure* variable*,* the mouth movement relevant variables have no unit and they should be synthesized with the registered scaling factor $\vec{c}$ and the 3D face model to yield the mouth *width* and *height* with a metric unit of millimetre. The curves in Figure 9 are the tracking results for a sequence of mouth movement. It shows that the estimated maximum mouth *width* and *height* value during the movement is 60 mm and 63 mm respectively while their manually measured values are 58 mm and 65 mm, the corresponding errors are 2 mm and 2 mm.

The dark points on the curve of Figure 9 are sampled instances during the mouth movement procedure and their corresponding images overlaid with tracked mouth model are shown in Figure 10. The images show that the estimated lip tracks the real lip correctly for various mouth movements. Figure 9 and Figure 10 show that the proposed tracker could estimate the mouth movement with an accuracy of less than 2 mm.

### 5.3. Fitting to Different Persons

The framework estimates the following face shape parameters automatically to fit to different persons: brow vertical position, eye vertical position, eye width, eye height, eye separation, nose vertical position, mouth vertical position and mouth width. During implementation the pose, shape and animation parameters are optimized using an alternating strategy. Firstly the pose is optimized then the shape is optimized and finally the animation is optimized. The alternating strategy could reduce the coupling problem between the parameters and fit to different persons more accurately.

**Figure 11:**
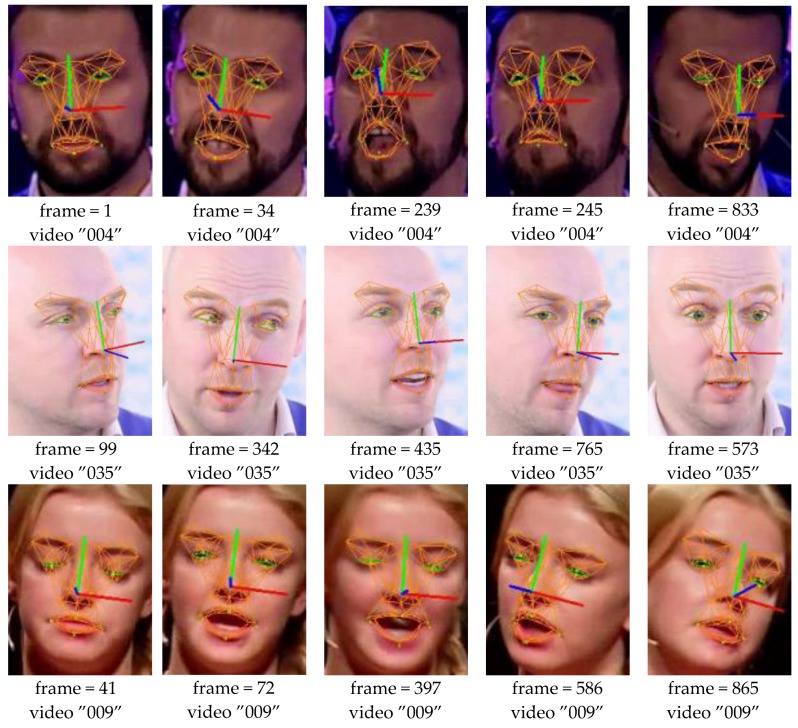
Tracked images of different persons from 300-VW database.

**Figure 12:**
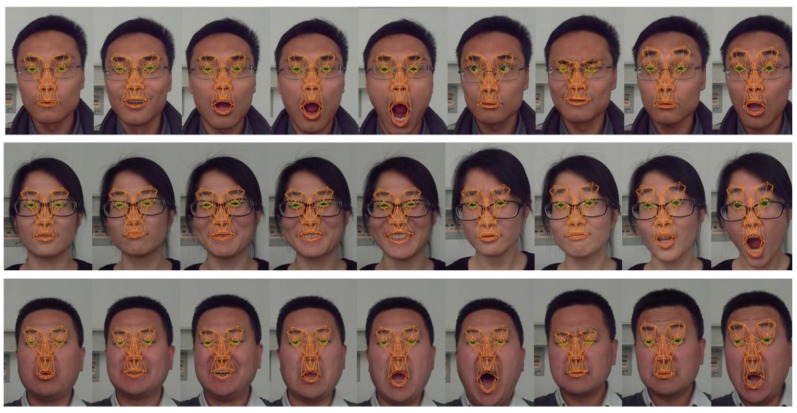
Tracked images of different persons in the laboratory.

Figure 11 gives some tracked images of different persons in the 300-VW database. It is shown that the framework could fit to different persons automatically and could estimate various expressions across poses. Figure 12 gives some tracked images of different persons in the laboratory. It shows that the framework could fit to different persons automatically and estimate various expressions, e.g., yawning, depression, smiling, surprising, talking.

## 6. Conclusions and Future Work

This paper develops a 3D facial movement tracking framework for real time human computer interface applications such as expression recognition, intention prediction, mental state estimation, etc. In such contexts, the 3D facial movement includes: (a) rigid global head movement and (b) non-rigid facial muscle movement. The proposed framework combines a DSR facial landmark localization module and an EKF 3D face tracking kernel. After comparing different DSR implementations quantitatively the framework decide to implement DSR with SIFT features and linear regression considering its accuracy and real time performance. An analytical EKF 3D face movement tracking kernel is derived, which could track the 3D face pose and animation parameters using 2D facial feature points. During implementation the pose, shape and animation parameters are optimized using an alternating strategy. Firstly the pose is optimized then the shape is optimized and finally the animation is optimized. The alternating strategy could reduce the coupling problem between the parameters and fit to different persons more accurately.

Experiments show that the proposed framework could track the face rotation with MAE of 4.23 (yaw), 5.65 (roll) and 2.36 (pitch) degree for BUFT datasets and 2.8 (yaw), 2.3 (roll) and 2.2 (pitch) degree for our laboratory datasets. The MAE of the eyelid distance tracking mainly locates within 1.2 pixels, while the MAE of the mouth width and height tracking mainly locate within 2.8 pixels on 300-VW database. Given registered face scale the framework could produce eyelid distance and mouth width/height with a metric of millimeter. The eyelid tracking error is 1 mm while the mouth width/height tracking error is 2 mm. Experiments on 300-VW and our laboratory dataset also show the framework could fit to different persons automatically and accurately thanks to the alternating optimizing strategy. 

In the future we will conduct research on the following aspects: increasing the working range of yaw rotation, enhancing the robustness against partial occlusion, pose-free gaze estimation and solving the variable coupling problem to increase the accuracy.

## Figures and Tables

**Table 1:** Mean absolute error of head pose estimation results on BUFT dataset.

Method	Yaw	Pitch	Roll	Mean
3D-Deform [32]	4.3	6.2	3.2	4.6
La Cascia et al. [30]	3.3	6.6	9.8	6.4
Sung et al. [33]	5.4	5.6	3.1	4.7
Valenti et al. [34]	6.6	6.4	4.2	5.6
Saragih et al. [15]	5.2	4.5	2.6	4.1
Ours	4.23	5.65	2.36	**4.08**

**Table 2:** Mean absolute error of head pose estimation for different testers.

Tester ID	Pitch				Mean MAE	
	[−15°, +15°]		[−25°, +25°]		[−15°, +15°]	[−25°, +25°]
	MAE	Frames	MAE	Frames		
#1	2.543°	188	2.562°	197	2.091°	2.2064°
#2	3.302°	163	3.953°	189		
#3	0.995°	109	1.062°	153		
#4	1.256°	138	1.379°	180		
#5	1.957°	217	1.700°	282		
#6	1.791°	63	2.973°	87		
**Tester ID**	**Yaw**				**Mean MAE**	
	**[−15°, +15°]**		**[−35°, +35°]**		**[−15°, +15°]**	**[−35°, +35°]**
	**MAE**	**Frames**	**MAE**	**Frames**		
#1	1.769°	173	2.300°	276	2.3559°	2.7898°
#2	2.962°	77	3.890°	145		
#3	2.037°	67	3.390°	124		
#4	2.866°	71	3.284°	194		
#5	2.519°	154	2.089°	308		
#6	2.710°	42	3.049°	82		
**Tester ID**	**Roll**				**Mean MAE**	
	**[−15°, +15°]**		**[−35°, +35°]**		**[−15°, +15°]**	**[−35°, +35°]**
	**MAE**	**Frames**	**MAE**	**Frames**		
#1	1.997°	132	1.957°	186	2.0344°	2.2981°
#2	2.888°	51	3.459°	94		
#3	2.084°	107	2.404°	157		
#4	2.878°	87	2.903°	168		
#5	1.302°	212	1.3024°	212		
#6	2.541°	75	2.708°	115		

**Table 3:** Mean absolute error (MAE) and standard deviation (STD) of eyelid and mouth tracking on 300-VW database. (The unit is pixel.)

Video ID	Pupil Dist.	Left Eyelid Dist.		Right Eyelid Dist.		Mouth Width		Mouth Height	
		MAE	STD	MAE	STD	MAE	STD	MAE	STD
“003“	58.7	0.8	0.7	0.8	0.7	2.1	1.5	1.8	1.6
“004“	59.2	0.8	0.9	0.9	0.8	1.9	1.5	**3.4**	2.1
“010“	67.9	0.9	1	1	1.1	1.5	1.2	1.8	1.5
“016“	95.8	0.5	0.5	0.5	0.4	3.6	2.7	2	1.7
“019“	74.8	0.7	1	0.6	0.8	**4.4**	2.5	1.8	1.5
“028“	69.2	0.3	0.3	0.4	0.4	2.1	1.5	1.3	1
“033“	107.8	1.2	1	**2.2**	1.1	2.5	1.7	2.4	2.1
“037“	82.5	0.4	0.3	0.4	0.4	2.2	1.6	**3.4**	1.8
“039“	116.9	0.4	0.4	0.5	0.4	2.5	2	2.4	2.1
“049“	49.9	0.7	0.6	0.8	0.6	1.2	1.8	1.9	2
“053“	73.7	0.6	0.3	0.4	0.3	1.3	1.1	1.8	1.2
“123“	101.1	**2.2**	1.3	1.3	1	1.6	1.1	3	1.7
“143“	78.1	1.8	0.7	1.9	0.7	3.8	1.9	1.9	1.2
“150“	72.1	1.2	0.6	1.1	0.6	1.4	1.1	0.9	0.8
“223“	110.6	1.6	1.4	1.6	1.4	2.6	1.9	2.4	1.9

## Supplementary Materials

The following are available online at http://www.mdpi.com/1424-8220/16/8/1157/s1, Video S1: Tracking results on videos “003” and “019” from 300-VW database.
